# Survey of Refuse Disposal Practices, Geohelminth Contamination, and Vector Abundance at Dumpsites in Akure Metropolis, Nigeria

**DOI:** 10.1155/japr/8298486

**Published:** 2025-12-21

**Authors:** O. S. Babatunde, I. A. Simon-Oke, I. I. Idowu, B. Adejoh, A. A. Olonisakin

**Affiliations:** ^1^ Department of Biology, Federal University of Technology, Akure, Ondo State, Nigeria, futa.edu.ng; ^2^ Department of Biology, Federal University Lokoja, Lokoja, Kogi State, Nigeria, fulokoja.edu.ng; ^3^ Department of Biotechnology and Molecular Biology, Federal University of Health Sciences, Ila-Orangun, Nigeria

**Keywords:** dumpsites, geohelminth, public health, refuse, vector, waste management, zoonotic

## Abstract

Rapid urbanization and poor waste management practices have led to environmental and public health concerns in Akure Metropolis, Nigeria. Open dumpsites serve as breeding grounds for vectors and reservoirs for soil‐transmitted helminths (STHs), increasing the risk of parasitic infections and vector‐borne diseases. This study investigates waste disposal practices, geohelminth contamination, and vector abundance at dumpsites to assess their implications for public health. The study was conducted at selected dumpsites in Akure Metropolis using a structured questionnaire to collect demographic and waste disposal data. Soil samples were collected and analyzed for helminth ova and larvae using the flotation and Bearmann culture techniques. Vectors, including insects and rodents, were captured and identified using standard entomological methods. Data analysis was performed using SPSS, with results presented in descriptive statistics and graphical formats. Out of 100 respondents, 30% used formal waste collection services, whereas 29% disposed of refuse directly at dumpsites, and 41% used both methods. Soil analysis revealed high contamination with *Strongyloides stercoralis*, *Ascaris lumbricoides*, and hookworm ova, with 14 out of 15 samples testing positive. Houseflies (*Musca domestica*), ants, mosquitoes, and rodents were abundant, acting as potential mechanical carriers of parasites. Ants were the most prevalent vectors (41.35%), followed by mosquitoes (30.77%) and houseflies (25.96%). Rodents (1.92%) were the least abundant. The study highlights the significant public health risks associated with refuse dumpsites due to high geohelminth contamination and vector proliferation. Urgent intervention is needed to improve waste management, public awareness, and sanitation practices to mitigate disease transmission risks in Akure Metropolis.

## 1. Introduction

Excessive waste generation and littering, driven by rapid population growth and urbanization, have become significant environmental concerns [1]. Waste production is closely linked to factors such as population size, industrialization, urbanization, lifestyle changes, and living standards [1–3]. In many developing countries, improper siting of waste disposal facilities remains a major challenge. Most dumpsites operate as open dumping systems due to the limited implementation of proper sanitary landfill technologies [4].

Dumpsites or landfills are designated areas for disposing of solid or semisolid waste generated from human and animal activities [1, 5]. However, inefficient waste management practices have resulted in a growing number of open dumps in Nigerian urban centers. Ikpeama et al. [5] further observed that solid waste continues to accumulate daily in Nigerian cities, worsening the waste management crisis.

As a result, these sites have become breeding grounds for flies, soil‐transmitted helminths, and other disease‐carrying vectors, posing significant health risks, obstructing traffic, and contributing to environmental degradation and visual pollution [6, 7]. Solid waste management in Nigeria faces challenges such as inefficient collection methods, limited coverage, and widespread illegal dumping [5, 6].

However, indiscriminate dumpsites in Akure Metropolis have raised serious concerns among residents due to uncontrolled refuse combustion, which often leads to fire outbreaks and hazardous smoke. Additionally, odours from decomposing waste degrade air quality, whereas street littering and drainage blockages are common issues. Studies indicate that selecting an appropriate waste disposal site is complex, requiring the evaluation of multiple criteria across various disciplines, including soil science, engineering, hydrogeology, topography, land use, sociology, and economics [5, 8]. Hence, it is necessary to evaluate the contamination of dumpsite soil by geohelminths and identify the vectors associated with dumpsites. This study is aimed at assessing waste disposal practices through a survey and to isolate and identify geohelminths and vectors present at dumpsites in Akure Metropolis.

## 2. Materials and Methods

### 2.1. Study Area

**Figure 1:**
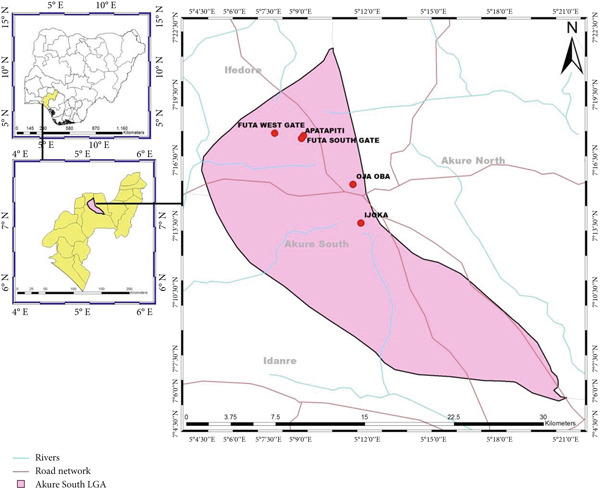
Map of Akure South LGA showing the study areas.

This study was conducted in Akure Metropolis, Ondo State, Nigeria, specifically within the Akure South Local Government Area (LGA), which lies between longitude 5° 81 ^′^ 20.651 ^″^ and latitude 7° 18 ^′^ 18.481 ^″^ in the tropical rainforest zone (Figure 1). The region experiences two distinct seasons: the rainy season (March to October) and the dry season (November to February), with temperatures ranging between 21°C and 28°C.

According to the 2006 National Population Census (NPC) [9], Akure South LGA had a population of 353,211, with an estimated annual growth rate of 3.03%.

The delineation of the study included five refuse dumpsites across both residential and commercial areas within Akure South LGA: Federal University of Technology, Akure (FUTA) West Gate, FUTA South Gate, Ijoka, Apatapiti, and the Oja‐Oba market area. These sites were purposively selected based on criteria such as high human activity, visible refuse accumulation, and local reports of poor sanitation and vector presence.

The map was generated using Esri ArcGIS Desktop 10.7.1 (Version 10.7.0.10450), based on GPS coordinates collected using a handheld GPS device (2kit Consulting, Düsseldorf, Germany, 2006–2013 Google Inc.). Software link: https://www.esri.com/en-us/arcgis/products/arcgis-desktop/overview. Two scale bars are included to improve orientation across both the main map and inset map for better spatial interpretation.

### 2.2. Demographic Data Collection

A structured questionnaire was used to collect demographic data. The questionnaire also gathered information on waste disposal practices, such as methods of household waste disposal, participation in environmental sanitation, availability of public waste collection systems, and the general state of refuse management in the communities.

### 2.3. Soil Sample Collection

Soil and vector collections were carried out once at each of the five selected dumpsites, using purposive sampling to select areas with active waste deposition, visible organic matter, and signs of frequent human or animal interaction.

Soil samples, approximately 100 g each, were collected from the selected dumpsites using a hand trowel at a depth of 2 cm. The samples were stored in clean, well‐labeled polythene bags and transported to the research laboratory at the Department of Biology, FUTA. All samples were analyzed within 24 h of collection and stored in a cool, dry place before examination.

### 2.4. Recovery of Ova Using the Floatation Technique

Helminth ova were recovered using a sodium chloride (NaCl) flotation technique with a specific gravity of 1.18, following the method described by Chatterjee [10]. A 50 g‐portion of soil was thoroughly mixed with 200 mL of distilled water in a Petri dish and then strained through a 150 *μ*m‐mesh sieve to remove coarse particles. The resulting filtrate was allowed to sediment in a glass beaker for 2 h. After decantation, the sediment was resuspended in 50 mL of distilled water and transferred into a centrifuge tube. The sample was centrifuged at 1500 rpm for 5 min, and the pellet was suspended in 15 mL of NaCl solution to facilitate flotation of helminth ova. The solution was carefully poured into a test tube to form a convex meniscus, over which a clean glass slide was placed for 3 min to allow eggs to adhere. The slide was then removed and examined under a compound microscope at ×10 and ×40 magnifications for the identification of helminth eggs.

This technique was employed for qualitative detection of helminth ova only. Quantitative egg counting methods, such as the Kato–Katz technique, were not applied, as the study was designed to assess the presence and diversity of geohelminth contamination at the dumpsites, rather than to determine egg intensity or burden.

Helminth ova were identified morphologically based on criteria described by Chatterjee [10], including egg shape, size, shell thickness, presence or absence of operculum, and internal embryonic structures, and were compared with standard diagnostic charts.

### 2.5. Recovery of Larvae Using the Modified Bearmann Culture Technique

The modified Bearmann′s method was employed for the extraction of larvae from soil samples. Funnels were filled with lukewarm water, and a weighed soil sample was placed on a white disposable paper towel secured with a rubber band, forming a pouch [7]. The pouch was suspended in the funnel filled with lukewarm water and left to stand for 72 h, allowing active larvae to migrate to the bottom of the funnel. The lower part of the suspension was collected in a universal bottle, and three drops of the sample were placed on a clean microscope slide for examination under a compound microscope Olympus CX23 compound microscope at ×10 and ×40 magnifications to detect the presence of larvae. Larval identification was based on morphological characteristics such as larval motility, buccal cavity length, and tail morphology, in accordance with the descriptions provided by Chatterjee [10].

### 2.6. Collection and Identification of Vectors

Insect vectors were identified under a dissecting microscope using the insect identification key by Choate [11], based on features such as wing venation patterns, antenna type, coloration, body segmentation, and leg morphology. Rodents (mice) were identified based on external features including body size, tail length relative to body, fur coloration, and ear shape, using general taxonomic characteristics referenced in vertebrate field identification guides.

## 3. Data Analysis

All collected data were recorded in Microsoft Excel and exported to IBM SPSS Statistics Version 26 for analysis. Descriptive statistics (frequencies, percentages) were used to summarize demographic data, waste disposal practices, and vector prevalence. Where applicable, Pearson′s chi‐square, phi coefficient, and Cramer′s *V* test were used to assess associations between categorical variables at a confidence level of *p* = 0.05. Graphical representations were created using Microsoft Excel. Inferential statistics such as odds ratios and confidence intervals were not calculated, as the study was not designed to measure exposure–outcome relationships.

### 3.1. Coordinate Collection and Map Construction

A study area map was constructed using Microsoft ArcGIS software (Esri ArcGIS Desktop 10.7.1, Version 10.7.0.10450, https://www.esri.com/en-us/arcgis/products/arcgis-desktop/overview).

## 4. Result

**Figure 2:**
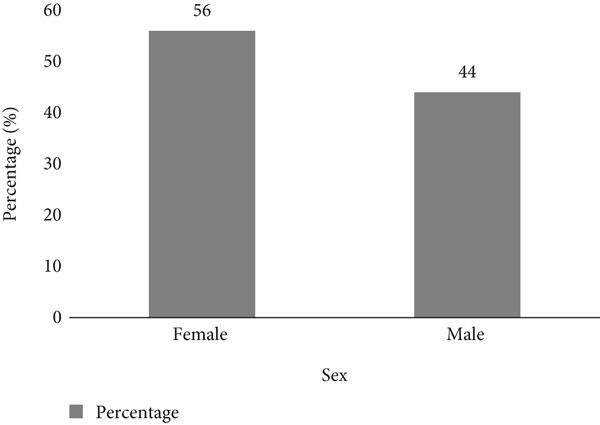
Gender distribution of respondents. This figure shows the gender composition of the survey respondents. Out of 100 respondents, 44% were male and 56% were female, indicating a nearly balanced participation in the study.

**Figure 3:**
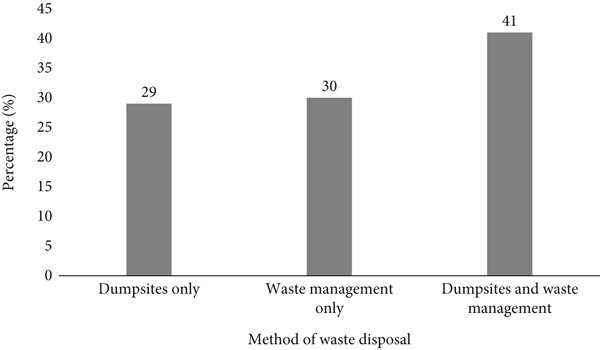
Waste disposal methods among respondents.Waste disposal practices among respondents illustrate that 30% relied solely on the waste management authority, 29% disposed directly at dumpsites, and 41% used both methods.

**Figure 4:**
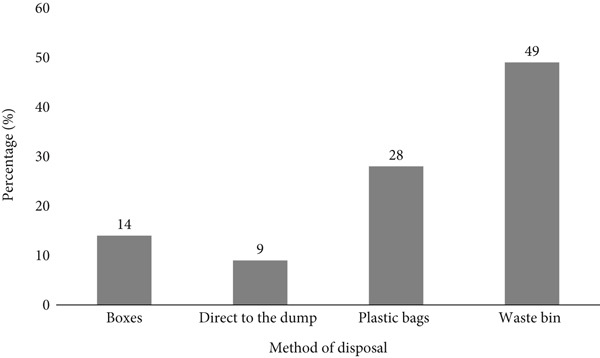
Methods of waste disposal among respondents using dumpsites. This figure illustrates the different methods used by respondents for waste disposal. Most respondents disposed of their waste in bins (49%), followed by plastic bags (28%), whereas fewer individuals used cardboard boxes (14%) or disposed of waste directly on dumpsites (9%).

A total of 100 questionnaires were distributed to residents near the surveyed dumpsites in Akure Metropolis, with 44% male (*n* = 44) and 56% female (*n* = 56) (Figure 2). The study examined waste disposal practices, showing that 30% of respondents relied solely on the waste management authority, 29% disposed of their waste directly at dumpsites, whereas 41% used both methods (Figure 3). The waste disposal methods also varied, with 14% using cardboard boxes, 9% disposing directly at dumpsites, 28% using plastic bags, and 49% disposing in bins (Figure 4). These findings highlight diverse waste disposal behaviours and indicate a reliance on both formal and informal waste management systems in the area.

Despite the reliance on waste management services, none of the respondents were aware of the specific methods used by the waste collection services for final disposal. These findings highlight the need for greater public awareness and improved waste management strategies in the area.

Table 1 presents the respondents′ knowledge of various diseases, indicating that 100% of participants were aware of waterborne and vector‐borne diseases. However, awareness of geohelminth infections was significantly lower, with 78% of respondents unaware of these diseases, 15% expressing awareness, and 7% having no knowledge of geohelminth infections.

**Table 1:** Respondents′ awareness of waste disposal methods, disease risks, and geohelminth infections in Akure Metropolis.

**Response**	**Awareness of method of disposal**	**Do you think media has helped to create awareness**	**Knowledge about water borne diseases**	**Knowledge about vector borne diseases**	**Knowledge about geohelminths**
Yes	100	95	100	100	15
No	0	3	0	0	78
No idea	0	2	0	0	7
Total	100	100	100	100	100

**Figure 5:**
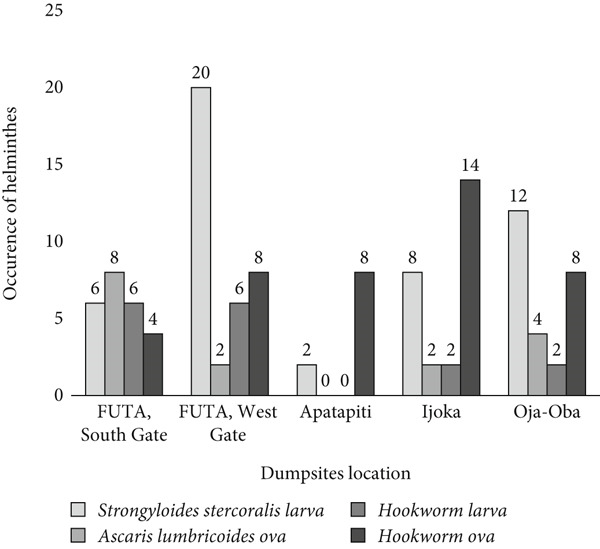
Distribution of isolated helminth parasites from the dumpsites. This figure shows the occurrence of helminth parasites recovered from the soil samples collected at dumpsites, indicating potential public health risks associated with improper waste disposal.

Figure 5 illustrates the prevalence of geohelminths across the sampled dumpsites. Of the 15 soil samples collected, 14 tested positive, resulting in a helminth contamination positivity rate of 93.3%, indicating a high level of parasitic presence in the refuse dumps. *Strongyloides stercoralis* larvae showed the highest occurrence, particularly at the FUTA West Gate dumpsite. This was followed by hookworm ova, which were also detected in 14 samples. *Ascaris lumbricoides* ova were most prevalent at the FUTA South Gate dumpsite, as shown in Figure 5.

**Figure 6:**
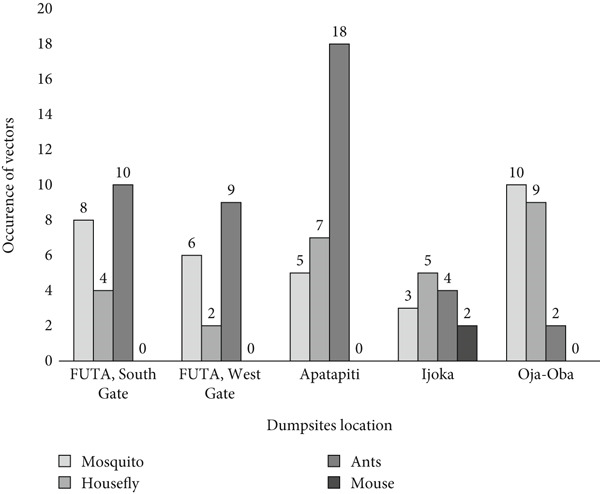
Distribution of isolated vectors from the dumpsites. This figure shows the prevalence of helminth parasites recovered from the soil samples collected at dumpsites, indicating potential public health risks associated with improper waste disposal.

Figure 6 presents the distribution of vectors across different dumpsites. Ants were the most frequently observed vectors, with the highest occurrence at the Apatapiti dumpsite. In contrast, two mice were identified at the Ijoka dumpsite, whereas the Oja‐Oba dumpsite recorded the highest mosquito population, suggesting favorable breeding conditions in that location.

A total of 122 parasite stages were recovered from the samples examined (Table 2). *S. stercoralis* larvae were the most frequently identified (39.34%), followed by hookworm ova (34.43%). In contrast, *A. lumbricoides* ova and hookworm larvae were less commonly observed, each accounting for 13.11% of the total recovery. Chi‐square analysis demonstrated a statistically significant difference in the distribution of parasite stages (*χ* ^2^ = 28.11, *d* *f* = 3, *p* < 0.001), indicating that the frequency of recovery was not uniform among the different parasite species and developmental forms.

**Table 2:** Parasite stages recovered and their relative abundance.

**Parasite stage/species**	**Number recovered**	**% abundance**
*Strongyloides stercoralis* (larva)	48	39.34
*Ascaris lumbricoides* (ova)	16	13.11
Hookworm (larva)	16	13.11
Hookworm (ova)	42	34.43
Total	122	100

*Note:* (*χ* ^2^ = 28.11, *d* *f* = 3, *p* < 0.001).

Table 3 summarizes the types and abundance of vectors collected from the dumpsites. The most dominant insect species were ants (41.35%), followed by mosquitoes (30.77%) and houseflies (25.96%). Rodents, represented by mice (1.92%), were the least abundant. The presence of these vectors underscores the potential public health risks associated with refuse dumps, as they serve as breeding grounds for disease‐transmitting organisms.

**Table 3:** Vectors identified on the dump sites.

**Vectors identified**	**Number collected**	**% abundance**
Mosquitoes	32	30.77
Houseflies	27	25.96
Ants	43	41.35
Mice	2	1.92
Total	104	100

*Note:* (*χ* ^2^ = 34.69, *d* *f* = 3, *p* < 0.001). This table presents the different vectors found at the dumpsites, including ants (41.35%), mosquitoes (30.77%), houseflies (25.96%), and mice (1.92%), emphasizing the role of these sites in vector‐borne disease transmission.

Chi‐square (*χ* ^2^) analysis revealed a statistically significant difference in the distribution of parasite stages recovered (*χ* ^2^ = 28.11, *d* *f* = 3, *p* < 0.001). *S. stercoralis* larvae were the most prevalent, whereas *A. lumbricoides* ova and hookworm larvae were less frequently recovered.

## 5. Discussion

The study examines waste disposal practices, environmental challenges, and health risks associated with refuse dumpsites in Akure Metropolis. It reveals that although 30% of residents have their waste collected by the Waste Management Board, 70% still use dumpsites, contributing to environmental pollution and health hazards [6]. Many respondents have large waste bins, but some still resort to direct disposal onto unmanaged dumpsites, which serve as breeding grounds for disease vectors and soil‐transmitted helminths.

Despite strong awareness of the health risks linked to living near dumpsites, many residents remain unaware of geohelminths, highlighting the need for education on these neglected tropical diseases (NTDs). This aligns with findings by Onyido et al. [1] and Oyewole and Simon‐Oke [7], emphasizing the link between improper waste management and public health concerns.

Several insect and rodent vectors were identified at the dumpsites, with houseflies and mosquitoes being the most abundant. Houseflies (*Musca domestica*) thrive in decomposing organic waste and are known carriers of zoonotic and nonzoonotic protozoan parasites [12, 13]. Mosquitoes, particularly *Anopheles* and *Aedes aegypti* species, pose a serious health risk by transmitting diseases such as malaria and dengue fever [8].

Ants, another prominent vector group, are capable of biting, stinging, and spraying irritant chemicals, which can cause mild to severe reactions in humans [14]. Their presence in human settlements and refuse dumpsites increases the risk of disease transmission. Rodents, particularly mice (*Mus musculus*) [15, 16], were also recorded, acting as carriers of hantavirus and salmonellosis, which can be spread through faecal matter, urine, saliva, or inhalation of contaminated dust [16]. These findings highlight the public health risks posed by unregulated waste disposal and the need for improved waste management and vector control measures.

The high prevalence of geohelminths in refuse dumpsites in Akure Metropolis poses a significant public health risk. Out of 15 soil samples collected, 14 tested positive for helminth contamination, with *S. stercoralis* being the most abundant, particularly at the FUTA West Gate dumpsite. These findings align with previous studies identifying refuse dumps as reservoirs of helminth infections, especially in areas with poor waste management [7].

Geohelminths thrive in unsanitary environments and can be transmitted through contaminated soil, food, water, or direct skin contact. *A. lumbricoides* and hookworms are linked to malnutrition, anaemia, and gastrointestinal infections, particularly in populations with poor hygiene and inadequate sanitation [7, 17]. These international reports underscore that geohelminth contamination is a global environmental health concern, with similar patterns observed in both tropical and temperate regions, reinforcing the need for sustained monitoring and intervention in Nigeria.

The persistence of these parasites in dumpsites is due to ineffective waste disposal, favourable environmental conditions, and poor sanitation practices [8]. Given that most respondents were unaware of geohelminth‐related diseases, public health education is necessary to promote proper waste disposal and hygiene practices, reducing the risk of infections in affected communities [1, 13, 17, 18].

The study highlights the role of vectors such as houseflies, ants, and rodents in transmitting geohelminths from dumpsites to human environments. Houseflies, being highly abundant, pick up helminth ova and larvae from contaminated soil and transfer them to food surfaces, facilitating the spread of parasites like *A. lumbricoides*, hookworms, and *S. stercoralis* [2, 12]. Similarly, ants and rodents act as mechanical carriers, contaminating food and water sources through their movement and faeces. This indirect transmission increases the risk of intestinal parasitic infections, especially in areas with poor sanitation and inadequate food hygiene, posing a significant public health concern.

Continuous environmental monitoring and periodic parasitological surveys are crucial for assessing the effectiveness of waste management and public health interventions. Such long‐term evaluations will help determine whether control measures reduce helminth contamination and vector abundance over time, ultimately improving community health outcomes.

## 6. Conclusion

This study assessed five refuse dumpsites in Akure Metropolis and found that they serve as reservoirs for soil‐transmitted helminths, such as *A. lumbricoides* and hookworms. In addition to the health risks posed by helminth contamination, these dumpsites provide an ideal breeding ground for vectors of infectious diseases, including houseflies, mosquitoes, and rodents. These vectors are known carriers of pathogens that cause typhoid fever, malaria, dysentery, dengue fever, and other infectious diseases, posing significant public health risks.

A key strength of this study is its integrated approach, combining environmental parasitology, entomological surveillance, and community‐level data, which provides a holistic understanding of health risks at refuse dumpsites. However, the study also has limitations. Soil and vector samples were collected only once, limiting the assessment of seasonal variation. Additionally, egg counting was qualitative, and molecular identification of parasites or vectors was not conducted due to resource constraints. Despite these limitations, the findings remain relevant for guiding public health interventions, particularly in urban and peri‐urban settings with poor waste management.

Given these findings, urgent intervention by relevant government agencies is necessary to mitigate the environmental and health hazards posed by open refuse dumps. Without prompt action, there is a potential risk of epidemics arising from continued exposure to these unsanitary conditions.

## Ethics Statement

This study was conducted following ethical guidelines, with verbal informed consent obtained from all participants involved in the questionnaire survey.

## Consent

Consent was secured from homeowners residing near the dumpsites before data collection. The study ensured confidentiality and voluntary participation, with no personally identifiable information recorded.

## Disclosure

This manuscript has been reviewed by all authors.

## Conflicts of Interest

The authors declare no conflicts of interest.

## Author Contributions

**O. S. Babatunde:** Conceptualization, validation, visualization, writing—review and editing. **I. A. Simon-Oke:** Investigation, visualization, data curation, writing—review and editing. **I. I. Idowu:** Methodology, data acquisition, writing—original draft. **B. Adejoh:** Conceptualization, data analysis, writing—review and editing. **A. A. Olonisakin:** Data analysis, writing—review and editing.

## Funding

No funding was received for this manuscript.

## Data Availability

The data of this study will be provided by the corresponding authors upon request.

## References

[1] Onyido A. E. , Azubuike J. , Amadi E. S. , Obiukwu M. O. , Ozumba N. A. , and Ikpeze O. O. , A Survey of Public Health Disease Vectors Breeding in Refuse Dumps in Onitsha Metropolis, Anambra State Nigeria, New York Science Journal. (2011) 4, no. 9, 34–39.

[2] Sangkachai N. , Gummow B. , Hayakijkosol O. , Suwanpakdee S. , and Wiratsudakul A. , A Review of Risk Factors at the Human-Animal-Environmental Interface of Garbage Dumps that Are Driving Current and Emerging Zoonotic Diseases, One Health. (2024) 19, 100915, 10.1016/j.onehlt.2024.100915, 39468997. 39468997 PMC11513544

[3] Zhang Z. , Chen Z. , Zhang J. , Liu Y. , Chen L. , Yang M. , Osman A. I. , Farghali M. , Liu E. , Hassan D. , Ihara I. , Lu K. , Rooney D. W. , and Yap P. S. , Municipal Solid Waste Management Challenges in Developing Regions: A Comprehensive Review and Future Perspectives for Asia and Africa, Science of the Total Environment. (2024) 930, 172794, 10.1016/j.scitotenv.2024.172794, 38677421. 38677421

[4] Olukanni D. , Akinyinka O. , Ede A. , Akinwumi I. , and Ajanaku K. , Appraisal of Municipal Solid Waste Management, Its Effect and Resource Potential in a Semi-Urban City: A Case Study, Journal of South African Business Research. (2014) 2014, 705695, 10.5171/2014.705695.

[5] Ikpeama C. A. , Obiajuru I. O. C. , and Ogomaka A. I. , The Impact of Refuse Disposal Dump Sites on the Spread of Intestinal Helminthiasis in Owerri Metropolis, IMO State, South Eastern Nigeria, International Journal of Clinical Chemistry and Laboratory Medicine. (2016) 2, no. 2, 13–18, 10.20431/2455-7153.0202003.

[6] Abubakar I. R. , Maniruzzaman K. M. , Dano U. L. , AlShihri F. S. , AlShammari M. S. , Ahmed S. M. S. , Al-Gehlani W. A. G. , and Alrawaf T. I. , Environmental Sustainability Impacts of Solid Waste Management Practices in the Global South, International Journal of Environmental Research and Public Health. (2022) 19, no. 19, 10.3390/ijerph191912717, 12717. 36232017 PMC9566108

[7] Oyewole O. E. and Simon-Oke I. A. , Ecological Risk Factors of Soil-Transmitted Helminths Infections in Ifedore District, Southwest Nigeria, Bulletin of the National Research Centre. (2022) 46, no. 1, 10.1186/s42269-022-00700-8.

[8] Ikpeama C. A. , Obiajuru I. O. C. , Nwoke B. E. B. , and Ezike M. N. , A Survey of the Relative Abundance of Mosquitoes in Their Preferred Breeding Micro-Habitats in Refuse Dumps in Owerri, Imo State, Nigeria, Universal Journal of Agricultural Research. (2017) 5, no. 6, 323–328, 10.13189/ujar.2017.050601.

[9] (NPC), N. P. C. , Nigeria National Census: Population Distribution by Sex, State, LGAs and Senatorial District: 2006 Census Priority Tables (Vol. 3), 2006, Retrieved from http://www.population.gov.ng/index.php/publication/140-popn-distri-by-sex-state-jgas-and-senatorial-distr-2006.

[10] Chatterjee K. D. , Parasitology; Protozoology and Helminthology, 2009, 13th edition, CBS Publishers and Distributors Pvt. Ltd.

[11] Choate P. M. , Introduction to the Identification of Insects and Related Arthropods, Identifying Insects and Related Arthropods, 2003, 1–13, https://entnemdept.ufl.edu/choate/insectid.pdf.

[12] Otokunefor K. , Nwankwo P. C. , Nyema K. C. , and Agbagwa O. E. , Molecular Assessment of Resistance and Virulence Potential of VibrioSpecies Isolated From Dumpsites in Port Harcourt, Nigeria, Journal of Advances in Environmental Health Research. (2024) 12, no. 2, 102–107, 10.34172/jaehr.1333.

[13] Park R. , Dzialo M. C. , Spaepen S. , Nsabimana D. , Gielens K. , Devriese H. , Crauwels S. , Tito R. Y. , Raes J. , Lievens B. , and Verstrepen K. J. , Microbial Communities of the House Fly Musca Domestica Vary With Geographical Location and Habitat, Microbiome. (2019) 7, no. 1, 10.1186/s40168-019-0748-9, 31699144. PMC6839111 31699144

[14] Simothy L. , Mahomoodally F. , Neetoo H. , and Department of Agricultural and Food Sciences, Faculty of Agriculture, University of Mauritius, Réduit, Moka, 80837, Mauritius , A Study on the Potential of Ants to Act as Vectors of Foodborne Pathogens, AIMS Microbiology. (2018) 4, no. 2, 319–333, 10.3934/microbiol.2018.2.319, 31294218. 31294218 PMC6604928

[15] Kassegne K. , Zhou X. N. , and Chen J. H. , Editorial: Vectors and Vector-Borne Parasitic Diseases: Infection, Immunity, and Evolution, Frontiers in Immunology. (2021) 12, 729415, 10.3389/fimmu.2021.729415, 34367192. 34367192 PMC8335558

[16] Sarathy V. V. and Walker D. H. , Ideal Criteria for Accurate Mouse Models of Vector-Borne Diseases With Emphasis on Scrub Typhus and Dengue, The American Journal of Tropical Medicine and Hygiene. (2020) 103, no. 3, 970–975, 10.4269/ajtmh.19-0955, 32602433. 32602433 PMC7470543

[17] Ihnacik L. , Smigova J. , Soltys J. , Bobikova D. , Kuzevicova Z. , Kuzevic S. , Schusterova I. , and Papajova I. , The Survey of Soil-Transmitted Helminth Species Abundance in Slovakia With an Emphasis on Parameters Important for their Distribution, Frontiers in Medicine. (2022) 9, 1043313, 10.3389/fmed.2022.1043313, 36465912. 36465912 PMC9712972

[18] Tadege B. , Mekonnen Z. , Dana D. , Sharew B. , Dereje E. , Loha E. , Verweij J. J. , Casaert S. , Vlaminck J. , Ayana M. , and Levecke B. , Assessment of Environmental Contamination With Soil-Transmitted Helminths Life Stages at School Compounds, Households and Open Markets in Jimma Town, Ethiopia, PLOS Neglected Tropical Diseases. (2022) 16, no. 4, e0010307, 10.1371/journal.pntd.0010307, 35377880. 35377880 PMC9009776

